# Supraclavicular block with Mepivacaine vs Ropivacaine, their impact on postoperative pain: a prospective randomised study

**DOI:** 10.1186/s12871-021-01499-z

**Published:** 2021-11-09

**Authors:** Irén Sellbrant, Jon Karlsson, Jan G. Jakobsson, Bengt Nellgård

**Affiliations:** 1 Department of Anaesthesiology and Intensive Care, Institute of Clinical Science, Sahlgrenska Academy, University of Gothenburg, Sahlgrenska University Hospital, Gothenburg, Sweden; 2 Department of Orthopedic Surgery, Institute of Clinical Science, Sahlgrenska Academy, University of Gothenburg, Sahlgrenska University Hospital, Gothenburg, Sweden; 3 Department of Anaesthesia & Intensive Care, Institute of Clinical Science, Karolinska Institute, Danderyd University Hospital, Stockholm, Sweden

**Keywords:** Day surgery, Distal radial fracture, Local anaesthetics, Opioid consumption, Postoperative pain, Rebound pain, Supraclavicular plexus block

## Abstract

**Background:**

Supraclavicular block (SCB) with long-acting local anaesthetic is commonly used for surgical repair of distal radial fractures (DRF). Studies have shown a risk for rebound pain when the block fades. This randomised single-centre study aimed to compare pain and opioid consumption the first three days post-surgery between SCB-mepivacaine vs. SCB-ropivacaine, with general anaesthesia (GA) as control.

**Methods:**

Patients (*n* = 90) with ASA physical status 1–3 were prospectively randomised to receive; SCB with mepivacine 1%, 25–30 ml (*n* = 30), SCB with ropivacaine 0.5%, 25–30 ml (n = 30) or GA (n = 30) with propofol/fentanyl/sevoflurane. Study objectives compared postoperative pain with Numeric Rating Scale (NRS) and sum postoperative Opioid Equivalent Consumption (OEC) during the first 3 days post-surgery between study-groups.

**Results:**

The three groups showed significant differences in postoperative pain-profile. Mean NRS at 24 h was significantly lower for the SCB-mepivacaine group (*p = 0.018*). Further both median NRS and median OEC day 0 to 3 were significanly lower in the SCB-mepivacaine group as compared to the SCB-ropivacaine group during the first three days after surgery; pain NRS 1 (IQR 0.3–3.3) and 2.7 (IQR 1.3–4.2) (*p = 0.017*) and OEC 30 mg (IQR 10–80) and 85 mg (IQR 45–125) (*p = 0.004*), respectively. The GA-group was in between both in pain NRS and median sum OEC. Unplanned healthcare contacts were highest among SCB-ropivacaine patients (39.3%) vs. SCB-mepivacaine patients (0%) and GA-patients (3.4%).

**Conclusions:**

The potential benefit of longer duration of analgesia, associated to a long-acting local anaesthetic agent, during the early postoperative course must be put in perspective of potential worse pain progression following block resolution.

**Trial registration:**

NCT03749174 (clinicaltrials.gov, Nov 21, 2018, retrospectively registered).

**Supplementary Information:**

The online version contains supplementary material available at 10.1186/s12871-021-01499-z.

## Introduction

Distal radial fracture (DRF) is the most common fracture in the elderly, causing both suffering and substantial health care costs. A recent epidemiological study showed increasing incidences of DRF [1], with osteoporosis being a contributing factor [2]. Data from the Swedish fracture register shows that approximately 26% of all DRF’s are surgically treated and over the recent decade surgery has become increasingly common [3]. There are several options of anaesthesia for this surgical repair and regional anaesthesia, like supraclavicular block (SCB), has gained increasing popularity [4]. There is however sparse data on the protracted outcome, like recovery and the patients´ satisfaction per se*,* after various types of blocks [5].

Peripheral blocks are increasingly popular after the introduction of ultrasound-guided techniques thereby increasing the success rate. Pain is often prominent when the effects of a single shot peripheral block wears off. The experience of this phenomeneon noted at block resolution is usually refered to as “rebound pain” [6]. Several studies have high-lighted the risk for “rebound pain” when the regional anaesthesia wears off [7–12]. The incidence of “rebound pain” is unknown, but could reach 40% of patients after peripheral nerve block resolution [11]. Thus, prospective randomised studies are warranted relating to the anaesthetic technique for surgical repair of DRF in the elderly, particularly those treated in day surgery settings [13–15].

The aims of the present prospective randomised study were to compare pain and postoperative opioid consumption during the first three days following open surgical repair of DRF in patients receiving SCB-ropivacaine vs SCB-mepivacaine with general anesthesia (GA) as control.

The hypothesis´ to be tested was; a SCB-mepivacaine will be associated to a better postoperative pain progression and less postoperative opioid consumption compared to a SCB-ropivacaine following open surgical repair of DRF.

## Methods

### Ethics, consent and permissions. Consent to publish

This single-centre prospective randomised clinical trial was approved by the Gothenburg Ethical Committee May 31:st 2018, (registration number 214–18). It was also registered in the Sahlgrenska University Hospital GDPR (General Data Protection Regulation) database August 28, 2018. The study was conducted in accordance with the tenets of the 1964 Declaration of Helsinki. It was retrospectively registered in clinicaltrials.gov (NCT03749174) the 21:st November 2018 with explicit information about start of patient inclusion the third September 2018. All patients aged between 19 and 86 years, with ASA physical status 1–3, and scheduled for Day Surgery of a distal radius fracture between September third 2018 and June 15th 2020 at Department of Anaesthesia and Intensive Care, Sahlgrenska University Hospital/Mölndal Hospital, Gothenburg, Sweden, were assessed for eligibility. A written informed consent with permission to publish was obtained from all patients before enrolment. All data generated and analysed during the current study are available from the corresponding author on reasonable request.

All fracture-classification were made by an experienced orthopaedic surgeon. Opiod naïve patients were included when having a closed DRF, (Orthopaedic Trauma Association, assessed on radiographs and classified as AO 23 A-C1), ≤ 17 days from trauma and scheduled for operative fixation with a volar locked plate. Finally, maximum length-of-surgery had to be < 90 min and all surgeons used tourniquet. Exclusion criteria were; multifractures, pre-trauma inflammatory diseases, dementia, severe psychiatric disorder or cognitive dysfunction, ongoing drug/alcohol abuse, known local anaesthetic allergy, pregnancy and finally, no fluency of the Swedish language.

Totally 142 patients were eligible for study enclosure. Twenty-two patients declined study participation leaving 120 patient to be included following written informed consent. Further, 30 patients were recruited to another part of the study, (not a part of this analysis). Thus, 90 patients were randomised and included with 30 patients to one of three anaesthetic techniques using sequentially numbered opaque envelopes with a random allocation sequence in 2 blocks, by the investigator.

*Group 1:* Supraclavicular block (SCB) given as a singleshot: mepivacain 1%, 25–30 ml and iv sedation using propofol (*n* = 30). *Group 2:* SCB given as a singleshot ropivacain 0.5%, 25–30 ml and iv sedation using propofol (n = 30). *Group 3:* General anaesthesia (GA) using propofol/fentanyl/sevoflurane and laryngeal mask and no local anaesthesia (n = 30, control group.

All patients had open surgical repair with internal fixation by a senior orthopaedic surgeon. Postsurgery a dorsal plaster splint was applied and patients were immobilized 2 weeks postoperatively.

All patients received oral premedication; acetaminophen 1000 mg, oxycodone 5 or 10 mg (5 mg to > 70 year and/or < 60 kg), etoricoxib 90 mg (if no contraindication) and meclizine 25 mg. All patients were given betamethasone 8 mg *iv* early perioperatively before tourniquet was placed.

All SCB-blocks were performed by senior anaesthetists skilled in ultra-sound guided blockade technique. Blocks were placed under ultra-sound guidance with in-plane technique with goal to have local anaesthesia spread around the nerv-trunk. All SCB-block patients were offered a mild *iv* sedation with propofol perioperatively.

GA was induced in the operation theatre by an anaesthetic nurse and an anaesthesiologist. Anesthesia was induced with propofol and fentanyl and maintained with sevoflurane. Patients were given oxycodone *iv* (0.1 mg/kg) and received no additional local anaesthesia.

Patients were monitored in the Post-Anaesthesia Care Unit (PACU) until considered stable and adequately pain-relieved to be transferred to the step-down ward. SCB-patients, if sufficiently awake after sedation, by-passed the PACU and were taken directly to the step-down ward.

#### Data collection

All data were collected by the study nurse while patients were still in hospital and then by 3 follow-up telephone calls at 24, 48, 72 h after discharge. Data as; patient characteristics, NRS (0–10) for pain assessment at rest was performed; before surgery and 2, 24, 48, 72 h after surgery. NRS was also assessed at block resolution, when patients experienced full motor and sensory function.

NRS for nausea and vomiting were assessed at the same time-points.

Oxycodone consumption in hospital and after discharge, (the first 3 postoperative days), was collected. Oxycodone was iv administrated in PACU and orally administrated at the step-down unit and after discharge.

Perioperative observations were registered; time anaesthesia nurse was occupied with the patient, theatre time, surgery-time including plaster, PACU-time and time-to-discharge, (only day-surgery patients). Moreover, total SCB effect-time and effect-time after surgery was noted as well as unplanned admissions and healthcare contacts during the first postoperative week.

The patient obtained a protocol to note the type, dose and frequency of analgesic consumption at home and they all received the same postoperative pain management after discharge; oxycodone 5–10 mg and acetaminophen 1000 mg, respectively. (No NSAIDs or Coxibs was provided postoperatively.) They received a prescription of these medications to be taken ad libitum within a daily maximum dose of 30–40 mg oxycone and 4000 mg acetaminophen. All opioid analgesics were converted to opioid equivalents (mg of p.o. morphine). The specific conversion ratio used are shown in Additional file 1.

*Primary endpoint*; difference in pain (NRS) at rest at 24-h and further during the first three days after surgery between SCB performed with mepivacaine vs ropivacaine, with GA being control group.

*Secondary endpoints*; Postoperative Opioid Equivalent Consumption (OEC) during the first three postoperative days. Differences in pain and opioid consumption between SCB’s and GA controls were also analysed. Post Operative Nausea and Vomiting *(PONV)* and Post Discharge Nausea and Vomiting *(PDNV)* during the first 3 postoperative days. Perioperative events described above, number of patients by-passing PACU, number of unplanned admissions and unplanned health care contacts postoperatively were registered.

### Statistical analyses

Sample size calculation: The statistic was based on similar studies from public domain [8], (NRS 4 vs 6 with a standard deviation of 2). The power calculation gave us three groups of 25 patients with a power of 80% with a significance between groups at *p* < 0.05.

Continuous variables are presented as mean and standard deviation and medians and inter quartiale range (IQR) for skewed data and categorical variables, number of patients in percent (%). For comparison between independent T-test and ANOVA for normal distributed continuous variables and the Mann-Whitney U-test and Kruskal-Wallis test was used for skewed distributed continuous variables. Normal distribution was assessed by the Shapiro Wilk test. Chi-square and Fischer’s exact test was used for non-ordered categorical variables.

Primary outcome, difference in pain between SCB with ropivacaine vs mepivacaine, was assessed between groups mean NRS at 24 h and the median of the NRS values for the first three postoperative days for the analysis of pain pattern following resolution of the block (3 days NRS sum divided by 3). Opioid used was transposed into opioid equivalence in mg and median of oral opioid consumption for the first three days was used for analysis of opioid use difference between groups. Non-parametric test (Mann-Whitney U for two groups), was chosen for comparison of pain and OEC as Shapiro Wilk test for normal distribution was found significant for all NRS and OEC variables.

A *p* < 0.05 was considered statistically significant. All data was compiled into XL and statistical analyses were performed using SAS 9.4 (SAS Institute Inc., Cary, NC).

## Results

**Fig. 1:**
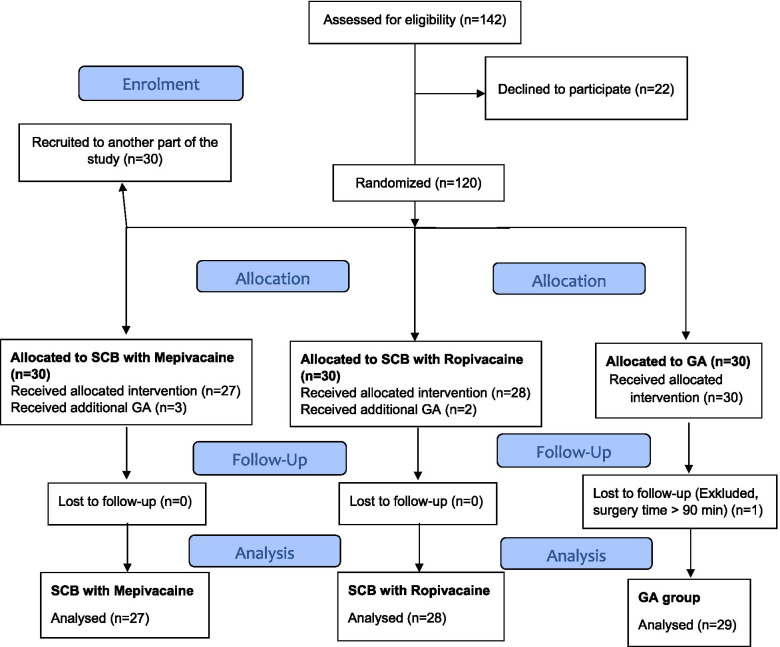
CONSORT Flowchart. Flow of patients through trial. *SCB* = supraclavicular block and *GA* = general anaesthesia

Nighty patients were initially recruited and 84 patients, 10 males/74 females, aged 19–86 years completed the protocol to assess primary and secondary outcomes, Flowchart Fig. 1.

Five patients, (2 in SCB-ropivacaine group and 3 in SCB-mepivacain group), were excluded due to failed blockade and therefore received GA. One patient (GA group) was excluded as surgery time exceeded 90 min.

The three groups were comparable in patients´ characteristics and preoperatively analgesic medication. There was only a slight difference in BMI between the 3 groups of patients studied, see Table 1.

**Table 1:** Patient characteristics. Patient characteristics and clinical data presented as mean (2 ± SD) or absolute number as appropriate

	SCB-mepivacaine (n = 27)	SCB-ropivacaine (***n*** = 28)	GA (***n*** = 29)	***p***-value
**Gender (male/female)**	3/24	3/25	4/25	0.93
**Age (yr)**	60.3 (±10.4)	62.3 (±13.1)	57.4 (±15.3)	0.51
**Height (cm)**	168.6 (±6.1)	168.2 (±6.9)	169.2 (±8.9)	
**Weight (kg)**	65.3 (±7.9)	70.8 (12.2)	72.6 (±13.1)	
**BMI (kg m**^**− 2**^**)**	22.9 (±2.3)	25.0 (±3.7)	25.3 (±3.9)	**0.03**
**Tobacco user:**				
**Smoking (yes/no)**	3/24	3/25	2/27	0.84
**Snuffing (yes/no)**	2/25	0/28	0/29	0.10
**ASA (1/2/3)**	13/13/1	9/19/0	9/20/0	0.32
**Days from injury to operation**	8.4 (±3.1)	10.0 (±4.2)	9.4 (±3.4)	0.27
**Injury to dominant hand (yes/no)**	8/19	11/17	11/18	0.72
**Apfel score before surgery and pain medication (1–4)**	3.07 (±0.62)	3.07 (±0.77)	3.31 (±0.85)	0.22
**Previous PONV/ history of motion sickness (yes/no)**	9/18	10/18	15/14	0.31

Classification of patients´ health and comorbidity level by the American Society of Anesthesiologists *(ASA)* system. Postoperative nausea and vomiting *(PONV)*, Body mass index *(BMI)*. Supraclavicular block *(SCB)*, General anaesthesia (*GA)*. Apfel score; riskfactors 1–4 for PONV

### Postoperative pain

**Fig. 2:**
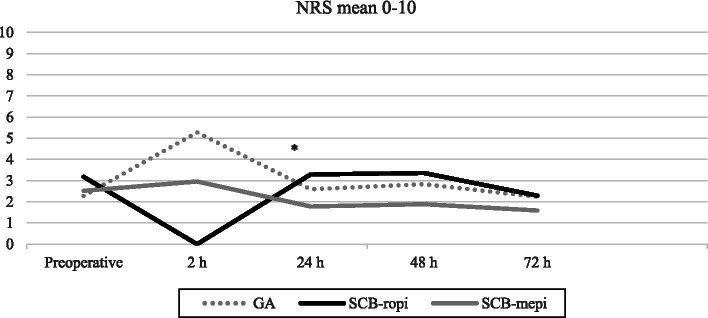
Mean NRS pain scores (range, 0–10) preoperative/baseline and four postoperative times are showing the postoperative pain pattern for the three anaesthetic groups. Postdischarge from hospital a differences between the 2 SCB-groups was noted at 24 h * (*p = 0.018*)

Pain ratings at base-line, preoperative mean NRS was similar between the 3 anaesthetic technique groups. There was a significant differences in postoperative pain profiles during the 3-days postoperative study period. The pain rating (mean NRS) at 24 h (following resolution of blocks) was significantly lower in SCB-mepivacaine group of patients (*p = 0.018*), see Fig. 2.

The median values for the first three postoperative days’ pain ratings (NRS) was significantly lower among the SCB-mepivacaine patients as compared to the SCB-ropivacaine group (*p* = 0.017). The GA-group median NRS was inbetween the SCB-ropivacaine and the SCB-mepivacaine groups, see Tables 2 and 3 and Fig. 2.

**Table 2:** Pain ratings for each assessment

Mean NRS (0–10)	SCB- mepivacaine (***n*** = 27)	SCB- ropivacaine (***n*** = 28)	GA (n = 29)
Preop	2.5 ± 1.8	3.2 ± 2.5	2.3 ± 2.1
2 h postop	3.0 ± 2.8	0	5.3 ± 2.2
24 h postop	1.8 ± 1.8	3.3 ± 2.7	2.6 ± 2.2
48 h postop	1.9 ± 2.1	3.4 ± 2.8	2.8 ± 2.1
72 h postop	1.6 ± 1.8	2.3 ± 2.2	2.2 ± 1.7
**Mean OEC (mg)**			
Before discharge (PACU+step-down unit)	10.4 ± 7.5	0.4 ± 1.9	18.8 ± 17.6
Postdischarge − 24 h	14.8 ± 15.3	33.2 ± 18.7	21.0 ± 14.5
24–48 h	13.7 ± 15.7	30.7 ± 21.1	17.9 ± 17.8
48–72 h	10.7 ± 14.7	19.6 ± 16.0	16.2 ± 15.7

Pain in mean (± 2 SD), NRS (0–10) and Opioid consumption in mean ± SD, OEC (mg). Opiod Equivalent Consumption *(OEC),* Numeric Rating Scale *(NRS).* SupraClavicular Block *(SCB).* General Anaesthesia *(GA)*

**Table 3:** Main postoperative outcome

	SCB- mepivacaine (*n* = 27)	SCB- ropivacaine (*n* = 28)	GA (*n* = 29)	*p*-value
Median mean NRS day 1–3	1 (0.3–3.3)	2.7 (1.3–4.2)	2.0 (1.3–3.0)	0.017
Median cumulative oral opioid use day 0–3 OEC mg	30 (10–80)	85 (45–125)	69 (20–90)	0.004

Main postoperative outcome, median mean NRS and median sum cumulative oral opioid use day 0–3, OEC mg; median and inter quartile range *(IQR).* Opiod Equivalent Consumption *(OEC),* Numeric Rating Scale *(NRS).* SupraClavicular Block *(SCB).* General Anaesthesia *(GA). p*-value assessed comparing mepivacaine and ropivacaine with Independent-Sample Median Test (Mann-W U)

The oral opioid consumption during the first three postoperative days was also significantly lower for the SCB-mepivacaine group compared to the SCB-ropivacaine group (*p* = 0.004) with the GA-group being in between see Table 3.

The SCB-ropivacaine had significantly longer duration of analgesia compared to SCB-mepivacaine.

The pain (NRS) experienced associated to the resolution of the local anaesthesia analgesia effect was similar between groups, Table 4.

**Table 4:** Perioperative time observations

Time events	SCB-mepivacaine (***n*** = 27)	SCB-ropivacaine (n = 28)	GA (n = 29)	***p***-value
**Anaesthesia nurse time (min)**	152.0 (±45.8)	146.8 (±40.4)	141.2 (±25.9)	0.61
**Theater time (min)**	191.9 (±47.6)	168.1 (±47.6)	186.8 (±37.4)	0.17
**Surgery + plaster time (min)**	71.1 (±20.6)	65.7 (±19.9)	69.8 (±16.4)	0.61
**PACU admitted patients (n,%)**	4 (14.8%)	1 (3.6%)	29 (100%)	
**Hospital time, DS patients (min)**	501 (±100)	501 (±78)	553 (±93)	0.08
**Overnight patients (n, %)**	2 (7.4%)	1 (3.6%)	1 (3.4%)	0.69
**Unplanned healthcare contacts** **the first postop week (n)**	0	11	1	
**Plexus block total duration time (hours)**	4.6 (±1.1)	18.9 (±5.2)	–	< 0.0001
**Plexus block duration time after surgery (hours)**	2.7 (±0.99)	16.4 (±5.1)	–	< 0.0001
**Mean NRS at plexus block resolution**	5.04 (±2.52)	4.86 (±3.34)	–	0.86

Data are presented as mean (2 ± SD) or for categorical data (n; %). Post Anaesthesia Care Unit (*PACU*), Numeric Rating Scale *(NRS)*, Day Surgery *(DS)*, Supraclavicular block *(SCB)* and General Anaesthesia *(GA)*

### Perioperative observations

Except for PACU-time, there were no differences in any other perioperative time events studied between study groups, Table 4.

All GA patients required PACU-observation with mean duration 114.7 (± 44.0) min. Only one SCB-ropivacaine and four SCB-mepivacaine patients needed PACU monitoring, mean 42 (±0) and 13.4 (± 35.4) min, respectively, Table 4.

Plexus block total duration time and remaining duration time after surgery, were significantly longer in the SCB-ropivacaine group, mean 18.9 and 16.4 h, respectively vs the SCB-mepivacaine group, mean 4.6 and 2.7 h, respectively (*p* < 0.0001). Pain, (mean NRS), at time when plexus block wore off did not differ between the two SCB-groups, Table 4.

### Unplanned admission and healthcare contacts the first week after surgery

Four (4.8%) patients were admitted overnight after surgery: 1 (3.4%) in the GA-group, 1 (3.6%) in the SCB-ropivacaine group and 2 (7.4%) in the SCB-mepivacaine group. The GA-patient was admitted because of pain, while the 3 SCB-patients were admitted as of social reasons, Table 4.

Twelve unplanned healthcare contacts were required during the first week after surgery; one in GA-group, 11 in SCB-ropivacaine group and none in SCB-mepivacaine group. The GA-patient (*n* = 1) contacted healthcare because of fever the first postoperative day. The SCB-ropivacaine patients contacted healthcare because of pain (*n* = 2), great discomfort with having a “dead arm” hours after surgery (n = 2) and one patient had a burn-damage on 2 fingers. (This latter patient touched a hot kettle with a still anaesthetized arm and needed repeated contacts (*n* = 6) because of blisters).

### PONV and PDNV (post discharge nausea and vomiting)

PONV and PDNV were consistently low and no significant differences between groups were seen at any time-point during the study period.

## Discussion

In this randomised study we found as expected, that a SCB with a single injection of the long-acting anaesthetic agent (ropivacaine), had a significantly longer duration compared to a SCB with a short-acting anaesthetic agent (mepivacaine). The most important finding was that the pain progression following resolution of the SCB’s was significantly worse in patients given ropivacaine compared to those given mepivacaine, both in median mean NRS and in the higher postoperative opioid consumption. Interestingly, the GA-patients show a median sum opioid consumption just in between the two SCB groups. Thus, GA-patients were not found to have worse pain scores the first three days after surgery compared to those given SCB’s. Finally, SCB-mepivacaine patients had no unplanned health care contacts after discharge during the 3-day follow-up, whereas SCB-ropivacaine patients had several visits.

The risk for rebound, i.e. worse pain at blockade resolution, in patients similar to ours, has been suggested by Galos et al. [8]. They found that a long-acting blockade resolved in the middle of the night at home, with a sometimes unmanageble pain as a result. Galos et al. [8] did however not compare different local anaesthetics with various resolution times and this initiated the present study. We wanted to investigate if pain, at blockade resolution, could superiorly be treated already in hospital after a short-acting blockade and see if this could result in less opioid consumption after discharge. We found this notion to be true in the present investigation.

Other randomised studies have shown a difference in experienced postoperative pain between GA and brachial plexus block with long-acting local anaesthetics for surgical treatment of DRF [5, 8, 16]. These studies found that GA patients experienced the highest NRS pain scores early after surgery, while patients in the brachial plexus group reported a delayed onset of pain. The present investigation confirms these results.

Previous studies have shown no difference in opioid consumption between GA and SCB, (with long-acting anaesthetic agents), during the first 3 postoperative days [5, 17]. The present study confirmed these results. Interestingly, during the first 3 postoperative days, the patients in the SCB-ropivacaine group had the highest total OEC while the SCB-mepivacine group had the lowest. Further, the SCB-ropivacaine patients needed almost no opioids prior to hospital discharge, but consumed most opioids later at home, while patients in the other 2 groups consumed most opioids during their hospital stay, monitored by hospital staff. This confirms our hypothesis to be true and may have clinical applications.

The findings of time-slots per se in perioperative period confirm those by Galos and colleagues [8], who also found no differences in surgical suite-time between GA and brachial plexus block groups, but a prolonged stay-period in PACU for GA patients. In the present study, all GA-patients stayed in PACU, while most SCB-patients could bypass the PACU and go directly to the stepdown unit. We found significant differences in the effective SCB block-duration between the two SCB-groups. Thus, the patients “experienced” SCB-duration from the block was administrated until it was totally worn off, was in mean 4.7 h in patients receiving SCB-mepivacaine block and 19.0 h in patients receiving SCB-ropivacaine. This sets a logistic demand to the perioperative management as the short-acting SCB may fade rapidly. Thus, the operation must start shortly after the SCB is administrated, otherwise chances enhance that *iv* opiods must be supplemented peri-operatively.

Sunderland and coworkers showed in 2016 that SCB-patients had a higher rate of unplanned healthcare contact because of pain compared to GA-patients for DRF-surgery [18]. We confirmed these findings in the present study where SCB-ropivacaine patients needed 11 unplanned health care contact the first week after surgery. Our patients contacted health care because of severe pain, unintended burn-damage or dissatisfaction of having a “dead arm” many hours after surgery.

Despite extensive information about the long-acting block, one patient visited the emergency department in the evening on day of surgery because of disconfort of the arm paralysis and one patient called us by telephone next day wanting hospital admission because of similar discomfort. Dissatisfaction because of long-acting motor block has been studied earlier [19], where the authors found no difference between long- and short-acting anaesthetic agent groups. However, in that study they used a mixture of long- and shortacting local anesthetics to give a mixture of central and periferal blockade aiming to prevent a time-wise long motor block.

### Strengths and limitations

This study had a prospective randomised clinical design. It was a single-centre design without any loss to follow-up reducing the risk of selection and information bias, and this warrants generalisability of this study. Investigator selection was avoided as only two investigators collected all data, ensuring consistency and a high standard of data collection. However, the trial was not blinded to any of the anaesthesia/surgery staff nor to the study nurse or the patients. We excluded patients with poor Swedish comprehension and severe pre-existing medical conditions and 22 patients declined to participate of different reasons. We only report on consumption of opioids and not on nonsteroidal anti-inflammatory drugs (NSAIDs) and acetaminophen. This is supported by results from a recent study [17] in which consumption of over-the-counter analgesics did not influence the mean OEC after open distal radial fracture surgery with use of either GA or SCB.

Our findings must naturally be put in perspective. Pain was over all low and differences between groups are significant different when compared for the period studied but the clinical diffrences may be argued. The low pain ratings must however be put in perspective of the significantly higher opioid use in the ropivacaine group. The groups were similar in demographics and all experienced pain prior to surgery. Surgery was performed by senior orthopeadic surgeons and all patients followed the same care pathway. Six patients in the SCB-groups were lost to follow-up, 5 because of failed block, insufficient block for surgery and subsequently was given GA in addition. One patient was lost because of prolonged surgery. Still the number in each group was in line with the power calculated numbers needed.

Future studies should explore if a short-acting local anaesthetic agent, proposedly with an adjuvant addition, in the SCB could make the initial postoperative recovery less painful and with less opioid consumption after discharge. It could also prevent unplanned healthcare contacts and make the recovery more safe and comfortable in day surgery. As pain is multidimensional, studies on pain should assess several outcome domains. Thus, future studies should include a more extensive evaluation of multidimensional pain-related patient reported outcomes, e.g. intensity of pain related to movement but also its interference with activities, side effects and perception of care [20, 21].

## Conclusion

Day surgery is expanding, including improved anaesthesia techniques, to facilitate safe and effective surgery and at the same time offering rapid, safe and effective recovery with a minimum of pain and other residual symptoms. In this study we found that the potential benefit of longer duration of analgesia, associated to the use of a long-acting local anaesthetic agent, during the early postoperative course must be put in perspective of potential worse pain course and a higher opioid consumption following resolution of the block after discharge from hospital. Thus, our hypothesis is confirmed that SCB with mepivacaine, a short-acting LA, is associated with less rebound pain. From our results we recommend short-acting local anesthetic agents to be used in SCB for surgical treatment of DRF to mitigate postoperative opioid-consumption and enhance patient comfort.

## Supplementary Information

**Additional file 1 **Equianalgesic conversion ratios. Oxycodone administered to patients in PACU and/or step-down unit and/or consumed by patients after discharge within the first three postoperative days were converted into mg of peroral (p.o.) Morphine. *i.v.* intravenous.

## Data Availability

All data generated and analysed during the current study are available from the corresponding author on reasonable request.

## Abbreviations

AO: Orthopaedic Trauma Association
ASA: American Society of Anesthesiologists
BMI: Body Mass Index
DRF: Distal Radial Fracture
DS: Day Surgery
GA: General Anasthesia
IQR: Inter Quartiale Range
NRS: Numeric Rating Scale
OEC: Postoperative Opioid Equivalent Consumption
PACU: Post-Anaesthesia Care Unit
PDNV: Post Discharge Nausea and Vomiting
PONV: Post Operative Nausea and Vomiting
SCB: Supraclavicular Block
