# Supplementary material for: Supraclavicular block with Mepivacaine vs Ropivacaine, their impact on postoperative pain: a prospective randomised study
Source: BMC Anesthesiol. 2021 Nov 9;21:273. doi: 10.1186/s12871-021-01499-z (PMC8577027; doi:10.1186/s12871-021-01499-z)
Supplement: Supplementary file 1 — Additional file 1 Equianalgesic conversion ratios. Oxycodone administered to patients in PACU and/or step-down unit and/or consumed by patients after discharge within the first three postoperative days were converted into mg of peroral (p.o.) Morphine. i.v. intravenous. [file 12871_2021_1499_MOESM1_ESM.docx]

**Appendix 1**

Equianalgesic conversion ratios. Oxycodone administered to patients in PACU and/or step-down unit and/or consumed by patients after discharge within the first three postoperative days were converted into mg of peroral (p.o.) Morphine. *i.v.* intravenous.

| **Type of opioid consumed** | **Conversion ratio to p.o. Morphine** |
| --- | --- |
| Oxycodone i.v. | 1:3 |
| Oxycodone p.o. | 1:2 |
